# Patterns of chromosomal translocations in Acute Leukemias: A Brazilian amazon perspective

**DOI:** 10.1016/j.htct.2026.106481

**Published:** 2026-06-12

**Authors:** Gláucia Lima Souza, Fabíola Silva Alves-Hanna, Joey Ramone Ferreira Fonseca, Andréa Monteiro Tarragô, Rafaella Oliveira Santos, Rechfy Kasem Abou Ali, Nadja Pinto Garcia, Maria do Perpétuo Socorro Sampaio Carvalho, Nelson Abrahim Fraiji, Adriana Malheiro, Allyson Guimarães Costa

**Affiliations:** a Diretoria de Ensino e Pesquisa, Fundação Hospitalar de Hematologia e Hemoterapia do Amazonas (HEMOAM), Manaus, AM, Brazil; b Programa de Pós-Graduação em Ciências Aplicadas à Hematologia, Universidade do Estado do Amazonas (UEA), Manaus, AM, Brazil; c Programa de Pós-Graduação em Imunologia Básica e Aplicada, Instituto de Ciências Biológicas, Universidade Federal do Amazonas (UFAM), Manaus, AM, Brazil

Dear Editor,

Acute leukemias (AL) are hematological malignancies characterized by the proliferation of progenitor cells in the bone marrow and peripheral blood. Chromosomal translocations represent the primary genetic alterations in these pathologies, serving as critical biomarkers for prognosis and therapeutic management [1]. Despite the high incidence of AL in the state of Amazonas, the frequency of these translocations in this specific population remained unknown.

Ninety patients were analyzed (59 with acute lymphoblastic leukemia [ALL] and 31 with acute myeloid leukemia [AML]) in this descriptive study conducted at the Fundação Hospitalar de Hematologia e Hemoterapia do Amazonas (HEMOAM) between 2021 and 2022. Reverse Transcription polymerase chain reaction (RT-PCR) following the BIOMED-1 Consortium protocol [2] was utilized to identify the most prevalent fusion transcripts. The results of this study revealed that 27 % of ALL patients presented translocations; *BCR*::*ABL1 p190* (8 %) and *ETV6*::*RUNX1* (8 %) were the most frequent (Supplementary Table 1). Among AML patients, the positivity rate was 42 %, with *RUNX1*::*RUNX1T1* (19 %) and *PML*::*RARA* (16 %) being the most frequent translocations (Supplementary Table 2).

The age and sociodemographic distribution exhibit regional particularities (Figure 1). In ALL, the *BCR*::*ABL1 p190* translocation was prevalent in adults (80 % of positive cases >18 years), while *ETV6*::*RUNX1* was concentrated in the pediatric group (60 % of cases <9 years) [3]. In AML, the *PML*::*RARA* translocation was also more frequent in adults (80 % >18 years), whereas *RUNX1*::*RUNX1T1* and *CBFB*::*MYH11* showed a more heterogeneous distribution across age groups (Supplementary Table 3). Overall, we observed a predominance of admixed individuals and residents of the capital, Manaus, reflecting the demographic reality and centralized access to health services in the Amazon region [4].

**Figure 1:**
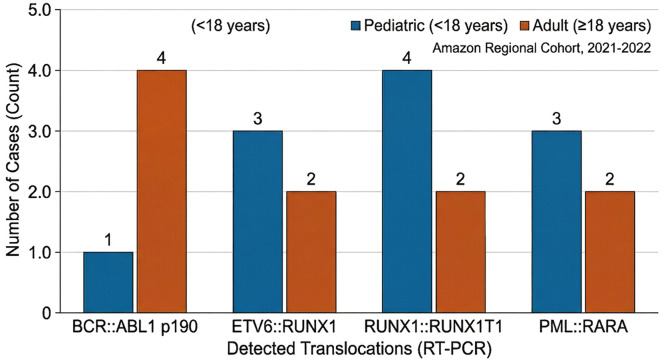
Frequency of major fusion transcripts in pediatric and adult acute leukemia cohorts from the Brazilian Amazon. The bar chart illustrates the frequency of major fusion transcripts detected by RT-PCR in a cohort of 90 patients (59 ALL and 31 AML) at the HEMOAM Foundation between 2021 and 2022. Data is stratified by age group: Pediatric (blue bars: <18 years) and Adult (terracotta bars: ≥18 years).

Clinically, the majority of patients (97 % ALL; 90 % AML) achieved complete remission. However, we highlight a rare case the of co-occurrence of *ETV6*::*RUNX1* and *KMT2A*::*AFF1* translocations in a pediatric patient, who died before the 35th day of treatment, reinforcing the impact of these alterations on clinical outcomes.

Although the sample size was limited by the impacts of the SARS-CoV-2 pandemic, this research establishes an unprecedented epidemiological profile for northern Brazil. The high prevalence of Pardo (admixed ancestry) individuals in our study aligns with the unique demographic mosaic of the northern region, which differs significantly from other Brazilian regions. While no genetic ancestry testing was performed, these sociodemographic findings suggest that regional population characteristics may influence the molecular landscape of acute leukemias. These data justify the implementation of routine molecular surveillance to optimize risk stratification and measurable residual disease monitoring in the Amazon, as recommended by the latest international guidelines.

## Conflicts of interest

The authors declare no conflicts of interest.

## Appendix Supplementary materials

Image, application 1

## Footnotes

Supplementary material associated with this article can be found in the online version at doi:10.1016/j.htct.2026.106481.
