# Supplementary material for: Patterns of chromosomal translocations in Acute Leukemias: A Brazilian amazon perspective
Source: Hematol Transfus Cell Ther. 2026 Jun 12;48(3):106481. doi: 10.1016/j.htct.2026.106481 (PMC13276133; doi:10.1016/j.htct.2026.106481)
Supplement: Supplementary file 1 [file mmc1.docx]

**Supplementary Table 1**: Summary of information specific to translocation-positive acute lymphoblastic leukemia patients

| **ID** | **Gender**  **/Age** | **Ethnicity** | **Residence** | **Infections** | **Family History** | **RG** | **Immunophenotype** | **Cytogenetics** | **WBC PB Day 0 (/mm^3^)** | **WBC PB Day 35 (/mm^3^)** | **BM Blasts Day 0**  **(%)** | **CNS Day 0** | **Infiltration** | **MRD**  **Day 35** | **Relapse** | **Death** | **CT** |
| --- | --- | --- | --- | --- | --- | --- | --- | --- | --- | --- | --- | --- | --- | --- | --- | --- | --- |
| 01 | M/42 | Indigenous | Manaus | None | ND | HR | B-ALL | ND | 31,930 | 761 | 46 | CNS-1 | Absent | Absent | Yes | No | *BCR*::*ABL p190* |
| 02 | F/41 | Admixed | Manaus | Varicella, Dengue, Measles and Hepatitis | Social alcoholism | HR | B-ALL | t(9;22)(q34.1;q11.2)[10] | 160,000 | 4,220 | 88 | CNS-1 | Absent | Absent | No | No | *BCR*::*ABL p190* |
| 03 | F/54 | ND | Manaus | None | ND | IR | B-ALL | t(9;22)(q34.1;q11.2), +der(22), +4, +5, +8, +17 | 7,660 | 13,720 | 80 | Absent | Absent | M2 | No | No | *BCR*::*ABL p190* |
| 04 | F/26 | ND | Manaus | None | ND | IR | B-ALL | 52, XX, t (9;22) (q34.1;q11.2), +der (22) t (9;22) (q34.1;q11.2), +4, +5, +8, +17 | 14,420 | 100 | 85 | CNS-1 | Adenomegaly | Absent | No | No | *BCR*::*ABL p190* |
| 05 | F/9 | ND | Manaus | None | ND | HR | B-ALL | ND | 157,900 | 2,880 | 37 | Absent | Absent | M2 | No | No | *BCR*::*ABL p190* |
| 06 | F/22 | Admixed | Manaus | Varicella | Stomach cancer (grandfather) | IR | B-ALL | ND | 8,750 | 2,850 | 88 | Absent | Absent | Absent | No | No | *BCR*::*ABL p190* |
| 07 | M/14 | Admixed | Manaus | None | ND | IR | B-ALL | 46, XY[5] | 182,000 | 1,470 | 90 | CNS-1 | Absent | M2 | No | No | *ETV6*::*RUNX1* |
| 08 | M/5 | ND | Barreirinha | None | ND | HR | B-ALL | 45, XY [5] | 30,300 | Absent | 73 | CNS-2 | Lymph node enlargement | Absent | Yes | Yes | *ETV6*::*RUNX1/*  *KMT2*::*AFF1* |
| 09 | M/12 | ND | Manacapuru | None | ND | HR | B-ALL | 46, XY [5] | 1,320 | 490 | 82 | Absent | Absent | M1 | No | No | *ETV6*::*RUNX1* |
| 10 | F/2 | Admixed | Manaus | None | ND | IR | B-ALL | ND | 100,000 | 7,370 | 85 | Absent | Absent | M1 | No | No | *ETV6*::*RUNX1* |
| 11 | M/6 | Admixed | Manaus | None | ND | IR | B-ALL | 46, XY [10] | 7,070 | 1,710 | 90 | CNS-1 | Absent | Absent | No | No | *ETV6*::*RUNX1* |
| 12 | F/20 | Admixed | Tabatinga | None | ND | IR | B-ALL | ND | 6,674 | 4,990 | 60 | CNS-1 | Absent | Absent | No | No | *TCF3*::*PBX1* |
| 13 | M/4 | ND | Itacoatiara | None | ND | LR | B-ALL | 46, XY, der(19)(1;19)(q23;p13.3)[10]/46, XY [10] | 62,720 | 2,750 | 88 | CNS-1 | Hepatosplenomegaly | Absent | No | No | *TCF3*::*PBX1* |
| 14 | F/45 | ND | São Gabriel da Cachoeira | Malaria | ND | HR | B-ALL | ND | 148,300 | 204,300 | 94 | Absent | Hepatosplenomegaly | Absent | No | No | *TCF3*::*PBX1* |
| 15 | M/4 | ND | Manaus | None | ND | HR | B-ALL | 4, XY, +4, +8, +9, +10, +13, +18, +20, +21,[4]/46, XY [16] | 2,190 | 5,880 | 60 | Absent | Absent | M1 | No | No | *KMT2*::*AFF1* |

F: Female; M: Male; ND: Not documented; RG: Risk Group; LR: Low Risk; IR: Intermediate Risk; HR: High risk; B-ALL: B Acute Lymphoblastic Leukemia; WBC PB: Peripheral blood White Blood Cell Count; CNS-1: Central Nervous System with count 0-5 blasts/µL; BM: Bone Marrow; CNS: Central Nervous System; CNS-2: Central Nervous System with count (5-15 blasts/µL); M1: <5% leukemic blasts cells; M2: 5-25% leukemic blast cells; MRD: Measurable residual disease; CT: Chromosomal translocation

**Supplementary Table 2:** Summary of information specific to translocation-positive acute myeloid leukemia patients

| **ID** | **Gender**  **/Age** | **Ethnicity** | **Residence** | **Infections** | **Family History** | **GR** | **Immunophenotype** | **Cytogenetics** | **WBC PB Day 0 (/mm^3^)** | **WBC PB Day 35 (/mm^3^)** | **BM Blasts Day0**  **(%)** | **CNS Day 0** | **Infiltration** | **MRD D35** | **Relapse** | **Death** | **CT** |
| --- | --- | --- | --- | --- | --- | --- | --- | --- | --- | --- | --- | --- | --- | --- | --- | --- | --- |
| 01 | F/28 | Admixed | Manaus | None | ND | HR | M2-AML | ND | 62,390 | 1,680 | 75 | CNS-1 | Visceromegaly | Absent | No | No | *RUNX1*::*RUNX1T1* |
| 02 | F/37 | Admixed | Manaus | None | ND | SR | AML | 46,XX,t(8;21)(q22;q22)[20] | 25,655 | 1,180 | 35 | CNS-1 | Absent | Absent | No | No | *RUNX1*::*RUNX1T1* |
| 03 | F/4 | Admixed | Manaus | None | ND | SR | AML | ND | 21,870 | 6,040 | 15 | CNS-1 | Hepatomegaly | Absent | No | No | *RUNX1*::*RUNX1T1* |
| 04 | M/15 | Admixed | São Gabriel da Cachoeira | None | ND | HR | M2-AML | 46, XY, t(8;21)(q22;q22)[20] | 25,480 | 5,110 | 74,4 | CNS-1 | Absent | M1 | No | No | *RUNX1*::*RUNX1T1* |
| 05 | F/12 | Admixed | Manaus | None | ND | HR | M2-AML | 46, XX, t(8;21)(q22;q22)[20] | 7,940 | 3,810 | 47 | CNS-1 | Absent | Absent | No | No | *RUNX1*::*RUNX1T1* |
| 06 | M/7 | Admixed | Juruá | None | ND | HR | AML | 46, XY, t(8;21)(q22;q22)[20] | 4,810 | 1,460 | 70 | CNS-1 | Cervical lymph node enlargement and mass in the right eye | M1 | No | No | *RUNX1*::*RUNX1T1* |
| 07 | M/12 | ND | Manaus | None | ND | HR | M3-AML | 46, XY[3] | 17,210 | 1,670 | 80 | CNS-1 | Gingival hyperplasia | Absent | No | No | *PML*::*RARA* |
| 08 | F/52 | ND | Manaus | None | Smoker and sporadic alcohol consumption | HR | M3-AML | 46,XX, t(15;17)(q24;q21) [16]/46,XX[4] | 1,910 | 1,270 | 70 | Absent | Adenomegaly | Absent | No | No | *PML*::*RARA* |
| 09 | F/76 | ND | Manaus | None | Former smoker | SR | AML | 53, X, +7, +8, +9, +10, +16, +19, +20 [18]/46,XX[2] | 47,020 | 3,450 | 70 | Absent | Absent | Absent | No | No | *PML*::*RARA* |
| 10 | M/42 | ND | Manacapuru | Dengue | Alcoholic e ex-addict (cocaine, 23-28 years) | - | AML | 46, XY, t15;17)(q24;q21)[15]/46, XY, del (1)(p35)[5] | 19,640 | - | 91 | Absent | Absent | Absent | No | No | *PML*::*RARA* |
| 11 | F/24 | Admixed | Manaus | None | ND | HR | M3-AML | ND | 510 | 2,560 | 90 | Absent | Absent | Absent | No | No | *PML*::*RARA* |
| 12 | M/4 | Admixed | Manaus | None | ND | HR | AML | 46,XY,inv(16)(p13;q22)[5] | 24,980 | 7,010 | 24 | CNS-1 | Adenomegaly | Absent | No | No | *CBFB*::*MYH11* |
| 13 | F/38 | Admixed | Silves | Measles, varicella e Mumps | Former alcoholic and cervical cancer (mother) | SR | AML | 46, XX[5] | 3,310 | 3,300 | 20 | CNS-1 | Absent | M1 | No | No | *CBFB*::*MYH11* |

F: Female; M: Male; ND: Not documented; RG: Risk Group; HR: High risk; SR: Standard Risk; AML: Acute Myeloid Leukemia; WBC PB: Peripheral blood White Blood Cell Count; CNS-1: Central Nervous System with count 0-5 blasts/µL CNS: Central Nervous System; CNS-2: Central Nervous System with count (5-15 blasts/µL); BM: Bone Marrow; M1: <5% leukemic blast cells; M2: 5-25% leukemic blast cells; MRD: Measurable residual disease; CT: Chromosomal translocation

| Variable | ALL | | | | | AML | | | | |
| --- | --- | --- | --- | --- | --- | --- | --- | --- | --- | --- |
|  | **Total** | *BCR*::*ABL p190* | *BCR*::*ABL p210* | *ETV6*::*RUNX1* | *TCF3*::*PBX1* | *KMT2*::*AFF1* | **Total** | *RUNX1*::*RUNX1T1* | *PML*::*RARA* | *CBFB*::*MYH11* |
|  | **n (%)** |  |  |  |  |  | **n (%)** |  |  |  |
| Age | **59 (100)** | **5 (8)** | **1 (2)** | **5 (8)** | **3 (5)** | **2 (3)** | **31 (100)** | **6 (19)** | **5 (16)** | **2 (6)** |
| <1 to 9 | 26 (44) | 1 (20) | - | 3 (60) | 1 (33) | 2 (100) | 8 (26) | 2 (33) | - | 1 (50) |
| >9 to <18 | 13 (22) | - | - | 2 (40) | - | - | 4 (13) | 2 (33) | 1 (20) | - |
| >18 | 20 (34) | 4 (80) | 1 (100) | - | 2 (67) | - | 19 (61) | 2 (33) | 4 (80) | 1 (50) |
| Gender | **59 (100)** | **5 (8)** | **1 (2)** | **5 (8)** | **3 (5)** | **2 (3)** | **31 (100)** | **6 (19)** | **5 (16)** | **2 (6)** |
| Male | 25 (42) | 1 (20) | 1 (100) | 4 (80) | 1 (33) | 2 | 11 (35) | 2 (33) | 2 (40) | 1 (50) |
| Female | 34 (58) | 4 (80) | - | 1 (20) | 2 (67) | - | 20 (65) | 4 (67) | 3 (60) | 1 (50) |
| Ethnicity | **39 (66)** | **3 (8)** | **1 (2)** | **3 (8)** | **3 (8)** | **2 (5)** | **22 (71)** | **5 (23)** | **1 (4)** | **2 (9)** |
| White | 6 (15) | - | - | - | - | - | - | - | - | - |
| Indigenous | 1 (2) | 1 (33) | - | - | - | - | - | - | - | - |
| Admixed (Pardo) | 32 (82) | 2 (67) | 1 (100) | 3 (100) | 1 (100) | - | 21 (95) | 5 (100) | 1 (100) | 2 (100) |
| Black | - | - | - | - | - | - | 1 (5) | - | - | - |
| Residence | **59 (100)** | **5 (8)** | **1 (2)** | **5 (8)** | **3 (5)** | **2 (3)** | **31 (100)** | **6 (19)** | **5 (16)** | **2 (6)** |
| Manaus | 38 (64) | 5 (100) | 1 (100) | 3 (60) | - | 1 (50) | 24 (77) | 4 (67) | 4 (80) | 1 (50) |
| Interior of Amazonas | 20 (34) | - | - | 2 (40) | 3 (100) | 1 (50) | 7 (23) | 2 (33) | 1 (20) | 1 (50) |
| Other states | 1 (2) | - | - | - | - | - | - | - | - | - |

**Supplementary Table 3:** Summary of demographic data of Acute Lymphoblastic Leukemia (ALL) and Acute Myeloid Leukemia (AML) patients treated at the HEMOAM Foundation

**Supplementary Table S4**: Summary of clinical data of Acute Lymphoblastic Leukemia (ALL) and Acute Myeloid Leukemia (AML) patients treated at the HEMOAM Foundation

| Variable | ALL | | | | | | | AML | | | |
| --- | --- | --- | --- | --- | --- | --- | --- | --- | --- | --- | --- |
|  | **Total**  **n (%)** | *BCR*::*ABL p190* | | *BCR*::*ABL p210* | *ETV6*::*RUNX1* | *TCF3*::*PBX1* | *KMT2*::*AFF1* | **Total**  **n (%)** | *RUNX1*::*RUNX1T1* | *PML*::*RARA* | *CBFB*::*MYH11* |
| Immunophenotype | **59 (100)** | | **5 (8)** | **1 (2)** | **5 (8)** | **3 (5)** | **2 (3)** | **31 (42)** | **6 (19)** | **5 (16)** | **2 (6)** |
| B-ALL | 57 (97) | | 5 (100) | 1 (100) | 5 (100) | 3 (100) | 2 (100) | - | - | - | - |
| Pre-B ALL | 1 (1,5) | | - | - | - | - | - | - | - | - | - |
| Pro-B ALL | 1 (1,5) | | - | - | - | - | - | - | - | - | - |
| M1 AML | - | | - | - | - | - | - | 2 (15) | - | - | - |
| M2 AML | - | | - | - | - | - | - | 5 (39) | 3 (50) | - | - |
| M3 AML | - | | - | - | - | - | - | 6 (46) | - | 3 (60) | - |
| WBC Count Day 0 | **59 (100)** | | **5 (8)** | **1 (2)** | **5 (8)** | **3 (5)** | **2 (8)** | **31 (100)** | **6 (19)** | **5 (16)** | **2 (6)** |
| <50.000/mm^3^ | 44 (75) | | 3 (60) | 1 (100) | 3 (60) | 1 (33) | 2 (100) | 24 (77) | 5 (83) | 5 (100) | 2 (100) |
| >50.000/mm^3^ | 15 (25) | | 2 (40) | - | 2 (40) | 2 (67) | - | 7 (23) | 1 (17) | - | - |
| WBC Count Day 35 | **47 (80)** | | **5 (11)** | **1 (2)** | **4 (8)** | **3 (6)** | **1 (2)** | **26 (84)** | **6 (23)** | **4 (15)** | **2 (8)** |
| <50.000/mm^3^ | 46 (98) | | 5 (100) | 1 (100) | 4 (100) | 2 (67) | 1 (100) | 26 (100) | 6 (100) | 4 (100) | 2 (100) |
| >50.000/mm^3^ | 1 (2) | | - | - | - | 1 (33) | - | - | - | - | - |
| Cytogenetics | **37 (63)** | | **3 (8)** | **-** | **4 (11)** | **1 (3)** | **2 (3)** | **22 (71)** | **4 (18)** | **4 (18)** | **2 (9)** |
| Unchanged karyotype | 15 (41) | | - | - | 4 (100) | - | 1 (50) | 8 (36) | - | 1 (25) | 1 (50) |
| Altered karyotype* | 22 (59) | | 3 (100) | - | - | 1 (100) | 1 (50) | 14 (64) | 4 (100) | 3 (75) | 1 (50) |
| Organ infiltration | **59 (100)** | | **5 (8)** | **1 (2)** | **5 (8)** | **3 (5)** | **2 (8)** | **31 (100)** | **6 (19)** | **5 (16)** | **2 (6)** |
| Yes | 26 (44) | | 1 (20) | - | 1 (20) | 2 (67) | 1 (50) | 11 (35) | 3 (50) | 2 (40) | 1 (50) |
| No | 33 (56) | | 4 (80) | 1 (100) | 4 (80) | 1 (33) | 1 (50) | 20 (65) | 3 (50) | 3 (60) | 1 (50) |
| CNS | **59 (100)** | | **5 (8)** | **1 (2)** | **5 (8)** | **3 (5)** | **2 (8)** | **14 (45)** | **6 (43)** | **5 (36)** | **2 (14)** |
| No | 55 (93) | | 2 (40) | 1 (100) | 2 (40) | 1 (33) | 1 (50) | 14 (100) | - | 4 (80) | - |
| CNS-1 | 1 (2) | | 3 (60) | - | 2 (40) | 2 (67) | - | - | 6 (100) | 1 (20) | 2 (100) |
| CNS-2 | 3 (5) | | - | - | 1 (20) | - | 1 (50) | - | - | - | - |
| Outcome | **59 (100)** | | **5 (8)** | **1 (2)** | **5 (8)** | **3 (5)** | **2 (8)** | **31 (100)** | **6 (19)** | **5 (16)** | **2 (6)** |
| Remission | 57 (97) | | 4 (80) | 1 (100) | 4 (80) | 3 (100) | 1 (50) | 28 (90) | 6 (100) | 5 (100) | 2 (100) |
| Relapse | 2 (3) | | 1 (20) | - | 1 (20) | - | 1 (50) | 3 (10) | - | - | - |
| Death | **59 (100)** | | **5 (8)** | **1 (2)** | **5 (8)** | **3 (5)** | **2 (8)** | **31 (100)** | **6 (19)** | **5 (16)** | **2 (6)** |
| Yes | 9 (15) | | - |  | 1 (80) | - | 1 (50) | 2 (6) | - | - | - |
| No | 50 (85) | | 5 (100) | 1 (100) | 4 (20) | 3 (100) | 1 (50) | 29 (94) | 6 (100) | 5 (100) | 2 (100) |

WBC PB: Peripheral blood White Blood Cell Count; CNS-1: Central Nervous System with count 0-5 blasts/µL CNS: Central Nervous System; CNS-2: Central Nervous System with count (5-15 blasts/µL).
